# Case Report: Bortezomib Plus CDOP Followed by Sequential Autologous Hematopoietic Stem Cell Transplantation and Lenalidomide-Based Maintenance Therapy in Plasmablastic Lymphoma

**DOI:** 10.3389/fmed.2021.749863

**Published:** 2021-12-03

**Authors:** Jiao Cai, Ling Qiu, Lei Ma, Nan Zhang, Fang-yi Fan

**Affiliations:** Department of Hematology and Hematopoietic Stem Cell Transplantation Centre, General Hospital of the Chinese People's Liberation Army Western Theater, Chengdu, China

**Keywords:** plasmablastic lymphoma, bortezomib, lenalidomide, autologous hematopoietic stem cell transplantation, ^18^F-FDG PET/CT

## Abstract

The standardized treatment plan for patients with plasmablastic lymphoma (PBL) remains controversial. Taking morphological characteristics and immunophenotypes into consideration may provide superior options for the treatment of PBL. In this case, we report that a myeloma-type regimen containing bortezomib plus cyclophosphamide, epirubicin, vindesine and prednisolone (CDOP) followed by sequential autologous hematopoietic stem cell transplantation (ASCT) and lenalidomide-based maintenance therapy to treat PBL may represent a promising regimen to improve the prognosis.

## Introduction

Plasmablastic lymphoma (PBL) is a highly aggressive and intractable subtype of B-cell-derived lymphoma that has a poor prognosis (1). Most cases of PBL occur in the oral cavity or jaw, gastrointestinal tract, lymph nodes, skin, and other sites, and they are closely related to factors such as dual infection with Epstein-Barr virus (EBV), human immunodeficiency virus (HIV), certain immunodeficiency-promoting factors, male sex, and advanced age (2–4). The typical immunophenotype of PBL is positive for the plasma cell markers CD79a, IRF-4/MUM-1, CD38, CD138, and BLIMP-1, but negative for the B-cell markers CD19, CD20, and PAX-5, and it is often detected with a high Ki-67 proliferation index (2, 4). Because of the low incidence of PBL, standard optimal therapeutic approaches have not been reported. The European Society for Medical Oncology (ESMO) guidelines recommend chemotherapy with CHOP treatment (5), while National Comprehensive Cancer Network (NCCN) guidelines recommend more intensive regimens, such as EPOCH, CODOX-M/IVAC, or hyper-CVAD therapy (6). Moreover, multiple studies have reported that the prognosis for patients with PBL is not improved by certain chemotherapy regimens compared with the CHOP program (7, 8). Since most patients are in the middle or late stages of diagnosis, patients with PBL show poor prognosis and a median overall survival (OS) of 8–12 months under traditional chemotherapy regimens (7, 9). Herein, we report on a 63-year-old male who presented with persistent left epistaxis. Enhanced MRI scanning revealed heterogeneous shadow filling in the left maxillary region. Immunohistochemical tests and ^18^F-FDG PET/CT results indicated stage II PBL. The patient was successfully treated with bortezomib plus CDOP, followed by sequential autologous hematopoietic stem cell transplantation, and lenalidomide-based maintenance therapy. The patient achieved complete response and durable remission with an OS of 32 months and a progression-free survival (PFS) of 22 months.

## Case Presentation

**Figure 1:**
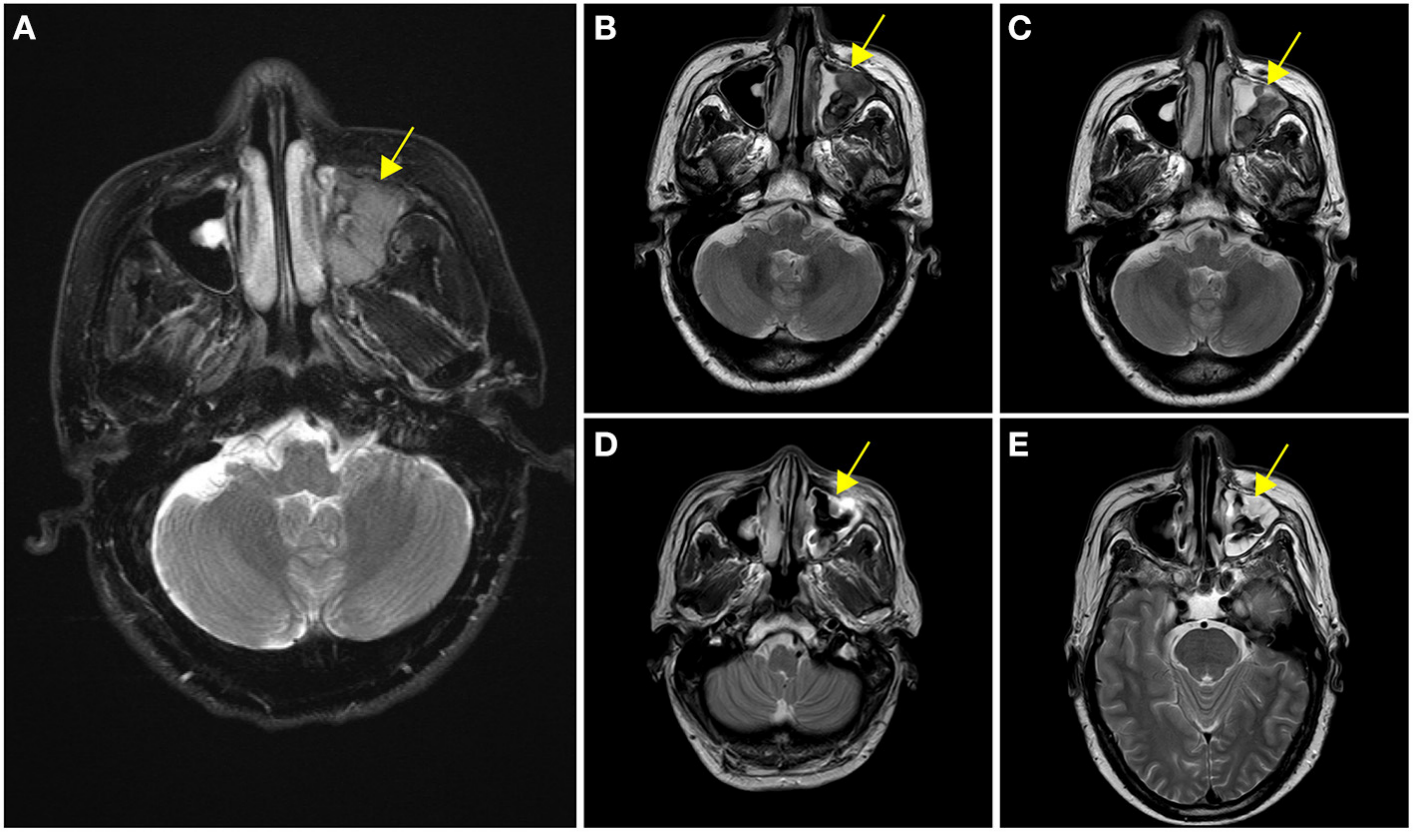
Enhancement of nasopharyngeal MRI scanning at different stages of treatment. **(A)** Initial enhancement MRI scanning revealed heterogeneous enhancement shadow filling in the left maxillary sinus at diagnosis. **(B)** After two cycles of V-CDOP chemotherapy, enhanced MRI scanning showed an excellent initial response with a substantial decrease in the size and intensity of nasopharyngeal lesions. **(C)** After four cycles of V-CDOP, MRI scanning revealed no significant change. **(D)** After radiotherapy and two cycles of RV-CDOP, subsequent enhancement MRI scanning revealed a reduction in the size of nasopharyngeal lesion. **(E)** Repeated enhancement MRI scanning after autologous hematopoietic stem cell transplantation (ASCT) showed increased abnormal signal shadows in the maxillary sinus, which was considered an inflammatory reaction. The nasopharyngeal lesions were indicated by yellow arrows.

A 63-year-old male presented on November 16, 2018, with complaints of persistent left epistaxis and a history of swelling in the left orbit and maxillary sinus for 3 months. Examination revealed multiple enlarged, indurated, and painless lymph nodes in the cervical and left submandibular areas, the largest of which was 4 mm × 3 mm. Blood tests at diagnosis showed anemia (Hb 122 g/L) and thrombocytopenia (90 × 10^9^/L). Syphilis and HIV screenings were negative. Initial enhancement MRI scanning on November 27, 2018, revealed heterogeneous enhancement shadow filling in the left maxillary sinus along with adjacent bone absorption (Figure 1A).

**Figure 2:**
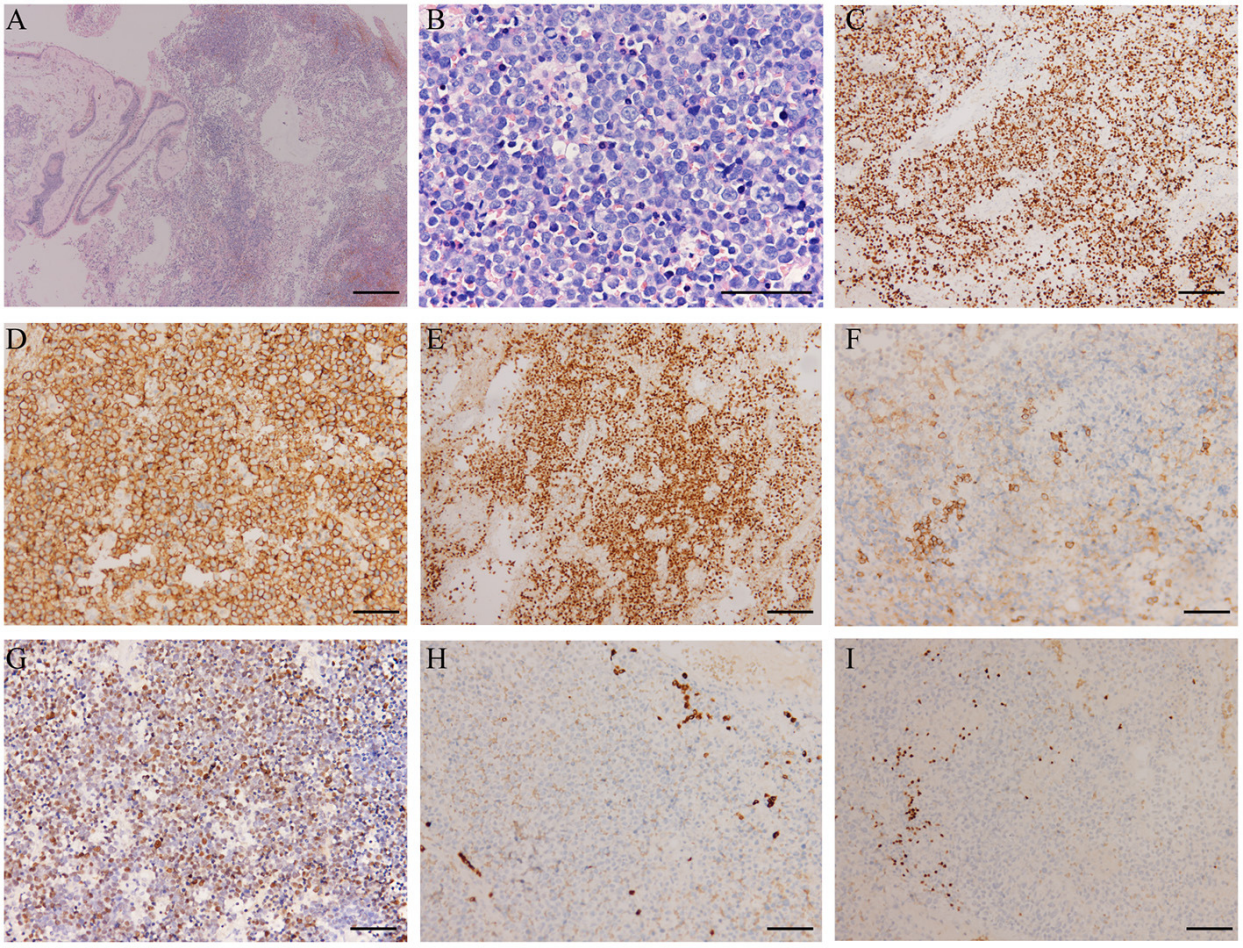
Histopathological features of the plasmablastic lymphoma (PBL) patient. **(A)** PBL consists of large atypical cells, which cause destroyed architecture (H&E; scale bar, 500 μm). **(B)** The higher power view shows large-sized atypical cells with lymphocytic or plasmacytoid morphology, accompanied by obvious nucleoli and pathological mitotic features (H&E; scale bar, 100 μm). **(C)** The proliferation index was high with a Ki-67 expression of almost 95% (scale bar, 200 μm). **(D)** The immunohistochemical profile showed the neoplastic cells were strongly positive for the plasma cells markers CD38 (scale bar, 100 μm) and **(E)** MUM-1 (scale bar, 200 μm), positive for CD138 (scale bar, 100 μm) **(F)**. **(G)** EBER *in situ* hybridization showed positive staining of plasmablasts (scale bar, 100 μm). The malignant cells did not express CD20 (scale bar, 100 μm) **(H)** and PAX-5 (scale bar, 100 μm) **(I)**.

**Figure 3:**
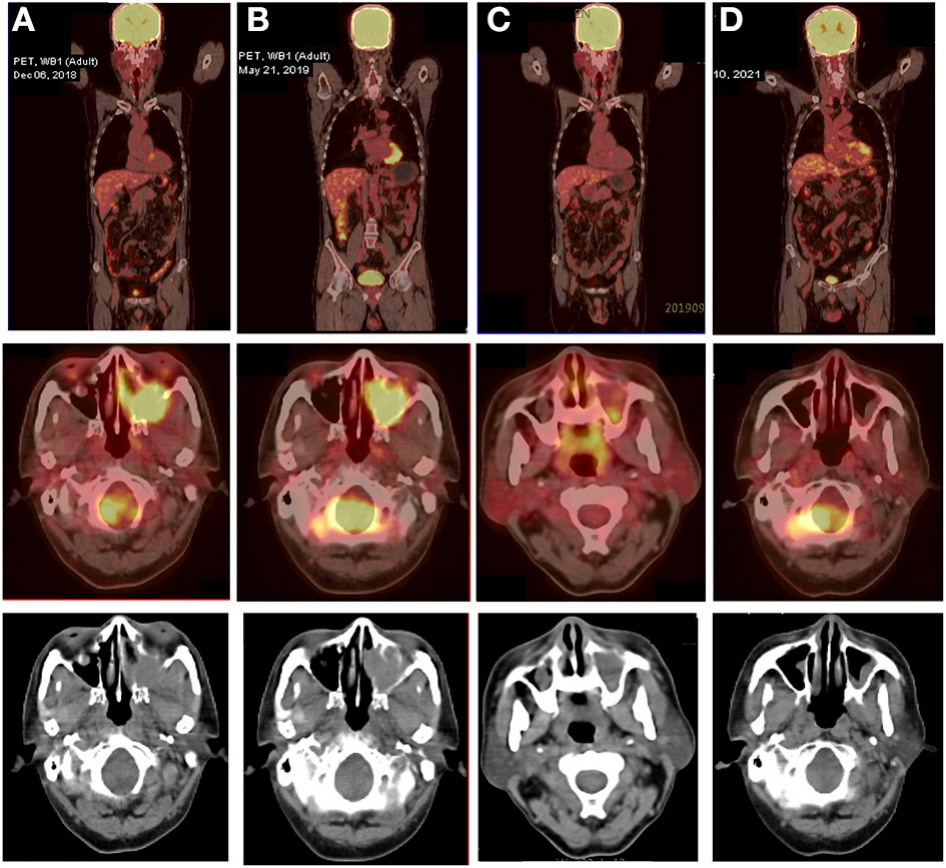
^18^F-FDG PET/CT images of the PBL patient at various stages of treatment. **(A)** Transverse PET/CT scan showed a markedly FDG-avid mass at the left maxillary sinus that invaded the left nasal cavity, left orbital apex, and left alar mandibular space at diagnosis (SUV_max_, 7.8; SUV_mean_, 6.3). **(B)** After five cycles of V-CHOP, re-evaluating PET/CT showed diminished range of lesions to 3.8 cm × 2.9 cm × 3.8 cm and reduced ^18^F-FDG uptake in the left maxillary sinus (SUV_max_, 7.0; SUV_mean_, 4.5) and other locations (SUV_max_, 4.0; SUV_mean_, 3.6). **(C)** After local radiotherapy and two cycles of RV-CDOP, PET/CT showed a complete metabolic response (cMR) with a diminished range of lesions to 1.0 cm × 1.3 cm and significantly reduced 18F-FDG uptake in the left maxillary sinus (SUV_max_, 3.5; SUV_mean_, 2.5) and a lack of metabolic activity in other regions. **(D)** Follow-up PET-CT 16 months post ASCT showed continued cMR.

The patient underwent a subsequent biopsy by nasal endoscopy on November 29, 2018. Histology highlighted a diffuse infiltrate of large atypical cells with lymphocytic or plasmacytoid morphology (Figures 2A,B). Neoplastic cells expressed a high proliferative index (Ki-67, 95%) (Figure 2C). Immunohistochemical profiling showed positive results in neoplastic cells for MUM1, CD38, CD138, c-myc, and EBV-EBER, the ratio of kappa chain (+) neoplastic cells to lambda chain (+) neoplastic cells is greater than 64:1 (Figures 2D–F), and they were negative for CD30, CD19, CD79a, PAX-5, CD20, CD21, bcl-6, CD10, bcl-2, CD15, HMB45, S-100, MelanA, CK, and EMA (Figures 2G–I).^18^F-FDG PET/CT was performed for further assessment of the mass lesion metabolic activity and the general conditions at diagnosis on December 6, 2018. Transverse PET/CT scan revealed a 3.7 cm × 3.9 cm × 4.3 cm solid mass, partial bone destruction, and swelling of soft tissues. A markedly FDG-avid space-occupying lesion was observed at the left maxillary sinus, and it invaded the left nasal cavity, left orbital apex, and left alar mandibular space (SUV_max_, 7.8; SUV_mean_, 6.3) (Figure 3A). Bone marrow aspirate and trephine biopsy were negative for lymphoma involvement. The screening investigations specific for multiple myeloma or plasmacytoma results showed that this patient was negative for serum protein electrophoresis, immunofixation, and free light chain assay. The final diagnosis was confirmed to be consistent with stage II PBL.

With curative intent, bortezomib was initiated (2.3 mg d1, 4, 8, 11) in combination with CDOP (cyclophosphamide 1.3 g d2, epirubicin 40 mg d2, vindesine 4 mg d21, prednisolone 65 mg d2–6) chemotherapy regimen on a 21-day cycle. After two cycles of V-CDOP, enhanced MRI scanning on February 11, 2019, revealed an excellent initial response, with a substantial decrease in the size and intensity of nasopharyngeal lesions (Figure 1B). After three cycles of the V-CDOP regimen, re-evaluation of the ^18^F-FDG PET/CT in the transverse dimension on May 21, 2019, showed a diminished range of lesions to 3.8 cm × 2.9 cm × 3.8 cm and reduced ^18^F-FDG uptake in the left maxillary sinus (SUV_max_, 7.0; SUV_mean_, 4.5) and other regions (SUV_max_, 4.0; SUV_mean_, 3.6) (Figure 3B). The patient achieved a partial response (PR) after five cycles of V-CDOP chemotherapy treatment.

This planned treatment was followed by mobilization and collection of autologous peripheral blood stem cells on May 24, 2019. After one cycle of V-CDOP, enhanced MRI scanning revealed no significant change on June 27, 2019 (Figure 1C). On regular follow-up, he received local radiotherapy (RT) to nasopharyngeal regions (30 Gy in 15 fractions over 3 weeks). After RT, he was switched to two cycles of V-CDOP regimens supplemented with lenalidomide (25 mg d1–21) on August 9 and September 10, 2019. The main toxic side effect was grade 2 myelosuppression and improved after symptomatic treatment. Subsequent enhancement MRI scanning revealed a reduction in the size of nasopharyngeal lesions on September 14, 2019 (Figure 1D). The therapeutic assessment by FDG PET/CT on September 29, 2019, showed a complete metabolic response (cMR), a diminished range of lesions to 1.0 cm × 1.3 cm, significantly reduced ^18^F-FDG uptake in the left maxillary sinus (SUV_max_, 3.5; SUV_mean_, 2.5), and a lack of metabolic activity in other regions (Figure 3C).

The patient received conditioning BEAM (BCNU, etoposide, cytarabine, and melphalan) high-dose chemotherapy with consolidative autologous hematopoietic stem cell transplantation (ASCT) in October 25, 2019. A total of 8.6 × 10^8^/kg mononuclear cells (MNCs) and 2.1 × 10^6^/kg CD34^+^ peripheral blood mononuclear cells (PBSCs) were reinfused, and complete engraftment was observed by day + 11. During autologous transplantation, he experienced grade 4 myelosuppression, requiring G-CSF. To consolidate the response, the patient received lenalidomide-based maintenance therapy after the transplant. A repeated enhancement MRI scanning showed increased abnormal signal shadows in the maxillary sinus, which was considered as an inflammatory reaction on May 6, 2020 (Figure 1E). He developed herpes zoster during maintenance treatment on October 2020. Follow-up PET-CT on March 10, 2021 showed continued cMR 16 months after ASCT (Figure 3D). Currently, the patient was monitored with serial MRI scanning and presented 32 months OS and 22 months PFS.

## Discussion

Plasmablastic lymphoma (PBL) has a poor prognosis, high malignancy, high recurrence rate, and more invasive features. The diagnosis of PBL is difficult because it has characteristics that overlap with myeloma and lymphomas (2). PBL is thought to be derived from plasmablasts, which are activated B cells that have undergone reactive processes, such as somatic hypermutation, EBV or HIV viral infections, and *MYC* gene rearrangements (2, 7). The morphologic characteristics of PBL are related to the location of the primary lesion (4). Cases of PBL arising in the oral cavity in HIV-positive patients display diffuse sheets of immunoblastic cells with abundant cytoplasm, vesicular chromatin, and prominent centrally placed nucleoli. Cases of PBL arising in lymph nodes and extranodal sites in HIV-negative patients tend to show apparent plasmacytic differentiation traits including nuclear pleomorphism, frequent mitosis pictures, and apoptosis bodies.

Multiple studies have confirmed that bortezomib, a proteasome inhibitor, has a definite therapeutic effect on multiple myeloma and can improve chemotherapy treatment in refractory or recurrent activated B cell-like (ABC) diffuse large B-cell lymphoma (10). In addition, bortezomib can slow down DNA replication and cell cycle progression by inhibiting NF-κB activity and downregulating MYC target genes and can play a synergistic role with other chemotherapeutic drugs, thus enabling patients with PBL to achieve better remission (8, 11–16). Because of the limited number of PBL cases, there is a lack of prospective randomized studies on the therapeutic effect of ASCT on PBL. Some small case series suggested that ASCT, after the first CR, was associated with improved remission in PBL and reinforced the treatment regimens, especially in refractory or relapsed PBL patients (7, 17–19). The immunomodulator, lenalidomide, has been widely used in the treatment of myeloma, and it has been used for the successful treatment of PBL, even refractory PBL, and can promote long-term remission states (20–22).

In our case, this PBL patient had multiple lesions of involvement at the left maxillary sinus that invaded the left nasal cavity, left orbital apex, and left alar mandibular space at presentation. Neoplastic histology showed characteristics of lymphocytic or plasmacytoid morphology, increased nuclear atypia, pathological mitosis, and a high proliferative index (Ki-67, 95%). Considering the aggressive features, our patient received six cycles of the V-CDOP chemotherapy regimens. After therapeutic assessment, he received local radiotherapy to nasopharyngeal regions and switched to two cycles of lenalidomide-added V-CDOP regimens. Follow-up PET-CT was performed with cMR. The patient was consolidated with ASCT after CR and received lenalidomide-based maintenance therapy after transplant. Currently, he remains in remission with a 32-month overall survival time and a 22-month progression-free survival time.

In summary, we selected a myeloma-type treatment regimen in consideration of the age, disease status, tolerance, and other factors of the patient, and showed that it may improve the outcomes of PBL patients. This case achieved complete response and durable remission after receiving bortezomib plus CDOP followed by sequential ASCT and lenalidomide-based maintenance therapy. However, the efficacy of the treatment regimen still needs to be validated by multi-institutional prospective therapeutic studies and larger sample sizes.

## Data Availability Statement

The raw data supporting the conclusions of this article will be made available by the authors, without undue reservation.

## Ethics Statement

Written informed consent was obtained from the patient for publication of this case report.

## Author Contributions

JC wrote the original draft and created the figures. LQ and F-yF were the major contributors in diagnosing and treating the patient. LM and NZ treated the patient. F-yF revised the manuscript. All the authors contributed to the manuscript revision, read, and approved the manuscript.

## Funding

We gratefully acknowledge the financial support by the Basic and Frontier Research Project of General Hospital of the Chinese People's Liberation Army Western Theater (Grant No. 2021XZYG-C44).

## Conflict of Interest

The authors declare that the research was conducted in the absence of any commercial or financial relationships that could be construed as a potential conflict of interest.

## Publisher's Note

All claims expressed in this article are solely those of the authors and do not necessarily represent those of their affiliated organizations, or those of the publisher, the editors and the reviewers. Any product that may be evaluated in this article, or claim that may be made by its manufacturer, is not guaranteed or endorsed by the publisher.

## References

[1] Jaffe ES. The 2008 WHO classification of lymphomas: implications for clinical practice and translational research. Hematol Am Soc Hematol Educ Prog. (2009) 2009:523–31. 10.1182/asheducation-2009.1.523 20008237 PMC6324557

[2] Castillo JJ Bibas M Miranda RN. The biology and treatment of plasmablastic lymphoma. Blood. (2015) 125:2323–30. 10.1182/blood-2014-10-567479 25636338

[3] Colomo L Loong F Rives S Pittaluga S Martínez A López-Guillermo A. Diffuse large B-cell lymphomas with plasmablastic differentiation represent a heterogeneous group of disease entities. Am J Surg Pathol. (2004) 28:736–47. 10.1097/01.pas.0000126781.87158.e3 15166665

[4] Harmon CM Smith LB. Plasmablastic lymphoma: a review of clinicopathologic features and differential diagnosis. Arch Pathol Lab Med. (2016) 140:1074–8. 10.5858/arpa.2016-0232-RA 27684979

[5] Hutchings M Ladetto M Buske C de Nully Brown P Ferreri AJM Pfreundschuh M. ESMO Consensus Conference on malignant lymphoma: management of ‘ultra-high-risk' patients. Ann Oncol. (2018) 29:1687–700. 10.1093/annonc/mdy167 29924296

[6] Horwitz SM Zelenetz AD Gordon LI Wierda WG Porcu P. NCCN guidelines insights: non-Hodgkin's lymphomas, Version 3.2016. J Natl Compr Cancer Netw. (2016) 14:1067. 10.6004/jnccn.2016.0117 27587620

[7] Castillo JJ Furman M Beltrán BE Bibas M Bower M Chen W. Human immunodeficiency virus-associated plasmablastic lymphoma: poor prognosis in the era of highly active antiretroviral therapy. Cancer. (2012) 118:5270–7. 10.1002/cncr.27551 22510767

[8] Loghavi S Alayed K Aladily TN Zuo Z Ng SB Tang G. Stage, age, and EBV status impact outcomes of plasmablastic lymphoma patients: a clinicopathologic analysis of 61 patients. J Hematol Oncol. (2015) 8:65. 10.1186/s13045-015-0163-z 26055271 PMC4472407

[9] Han X Duan M Hu L Zhou D Zhang W. Plasmablastic lymphoma: review of 60 Chinese cases and prognosis analysis. Medicine. (2017) 96:e5981. 10.1097/MD.0000000000005981 28248855 PMC5340428

[10] Dunleavy K Pittaluga S Czuczman MS Dave SS Wright G Grant N. Differential efficacy of bortezomib plus chemotherapy within molecular subtypes of diffuse large B-cell lymphoma. Blood. (2009) 113:6069–76. 10.1182/blood-2009-01-199679 19380866 PMC2699229

[11] Ando K Imaizumi Y Kobayashi Y Niino D Hourai M Sato S. Bortezomib- and lenalidomide-based treatment of refractory plasmablastic lymphoma. Oncol Res Treat. (2020) 43:112–6. 10.1159/000504608 31842017

[12] Castillo JJ Reagan JL Sikov WM Winer ES. Bortezomib in combination with infusional dose-adjusted EPOCH for the treatment of plasmablastic lymphoma. Br J Haematol. (2015) 169:352–5. 10.1111/bjh.13300 25612847

[13] Fedele PL Gregory GP Gilbertson M Shortt J Kumar B Opat S Grigoriadis G. Infusional dose-adjusted epoch plus bortezomib for the treatment of plasmablastic lymphoma. Ann Hematol. (2016) 95:667–8. 10.1007/s00277-016-2601-6 26801792

[14] Fernandez-Alvarez R Gonzalez-Rodriguez AP Rubio-Castro A Gonzalez ME Payer AR Alonso-Garcia A. Bortezomib plus CHOP for the treatment of HIV-associated plasmablastic lymphoma: clinical experience in three patients. Leukemia Lymphoma. (2016) 57:463–6. 10.3109/10428194.2015.1050666 25976108

[15] Dittus C Grover N Ellsworth S Tan X Park SI. Bortezomib in combination with dose-adjusted EPOCH (etoposide, prednisone, vincristine, cyclophosphamide, and doxorubicin) induces long-term survival in patients with plasmablastic lymphoma: a retrospective analysis. Leukemia Lymphoma. (2018) 59:2121–7. 10.1080/10428194.2017.1416365 29303024

[16] Castillo JJ Guerrero-Garcia T Baldini F Tchernonog E Cartron G Ninkovic S Cwynarski K. Bortezomib plus EPOCH is effective as frontline treatment in patients with plasmablastic lymphoma. Br J Haematol. (2019) 184:679–82. 10.1111/bjh.15156 29527667

[17] Lopez A Abrisqueta P. Plasmablastic lymphoma: current perspectives. Blood Lymphatic Cancer Targets Ther. (2018) 8:63–70. 10.2147/BLCTT.S142814 31360094 PMC6467349

[18] Liu M Liu B Liu B Wang Q Ding L Xia C. Human immunodeficiency virus-negative plasmablastic lymphoma: a comprehensive analysis of 114 cases. Oncol Rep. (2015) 33:1615–20. 10.3892/or.2015.3808 25695332 PMC4358079

[19] Tchernonog E Faurie P Coppo P Monjanel H Bonnet A Algarte Génin M. Clinical characteristics and prognostic factors of plasmablastic lymphoma patients: analysis of 135 patients from the LYSA group. Ann Oncol. (2017) 28:843–8. 10.1093/annonc/mdw684 28031174

[20] Bibas M Grisetti S Alba L Picchi G Del Nonno F Antinori A. Patient with HIV-associated plasmablastic lymphoma responding to bortezomib alone and in combination with dexamethasone, gemcitabine, oxaliplatin, cytarabine, and pegfilgrastim chemotherapy and lenalidomide alone. J Clin Oncol. (2010) 28:e704–e8. 10.1200/JCO.2010.30.0038 20823416

[21] Yanamandra U Sahu KK Jain N Prakash G Saikia U Malhotra P. Plasmablastic lymphoma: successful management with CHOP and lenalidomide in resource constraint settings. Ann Hematol. (2016) 95:1715–7. 10.1007/s00277-016-2732-9 27324386

[22] Schmit JM DeLaune J Norkin M Grosbach A. A case of plasmablastic lymphoma achieving complete response and durable remission after lenalidomide-based therapy. Oncol Res Treat. (2017) 40:46–8. 10.1159/000455146 28095384

